# Polypharmacy in the Treatment of Manic Episodes with Psychotic Features: A Two-Year Retrospective Analysis from the Psychiatric Clinic

**DOI:** 10.1192/j.eurpsy.2026.11196

**Published:** 2026-07-31

**Authors:** E. Prljača, E. Bećirović, A. Brigić, J. Hamidović, M. Mešanović

**Affiliations:** Department of Psychiatry, University Clinical Centar Tuzla, Tuzla, Bosnia and Herzegovina

## Abstract

**Introduction:**

Treatment guidelines for bipolar disorder generally recommend monotherapy or, at most, dual therapy for acute manic episodes. However, in real-world clinical practice, patients with mania and psychotic features often require more complex pharmacological strategies. Exploring this gap is important for tailoring treatment approaches.

**Objectives:**

To evaluate the frequency and patterns of polypharamcy in hospitalized patients with bipolar disorder, current episode mania with psyhotic features, over a two-year period at the Psychiatric Clinic, UKC Tuzla.

**Methods:**

We retrospectively analyzed medical documentation of patients diagnosed with mania with psychotic features who were hospitalized and treated during the study period. Data collected included gender distribution and prescribed pharmacotherapy

**Results:**

A total of 67 patients were included (43% male, 57% female). Treatment with two drugs, consistent with guideline recommendations, was observed in 31% of cases. However, polypharmacy was required in the majority: 34% of patients received three drugs, 26% received four drugs, and 9% were treated with five agents. The most frequent regimens combined a mood stabilizer with one or more antipsychotics, often supplemented with benzodiazepines. Importantly, polypharmacy was mandatory in most cases to achieve stabilization, particularly in patients with severe psychotic symptoms, pronounced psychomotor agitation, and potential aggressive behavior.

**Conclusions:**

Despite guidelines advocating for monotherapy or dual therapy, polypharmacy was often unavoidable in treating mania with psychotic features. While effective for stabilization, such regimens may be associated with increased risk of side effects and adherence challenges. These findings underscore the complexity of real-world clinical practice and highlight the need for future guidelines to better reflect treatment strategies for patients presenting with severe symptoms.

**Disclosure of Interest:**

None Declared

